# Gastro-oesophageal reflux: a mixed methods study of infants admitted to hospital in the first 12 months following birth in NSW (2000–2011)

**DOI:** 10.1186/s12887-018-0999-9

**Published:** 2018-02-12

**Authors:** Hannah Grace Dahlen, Jann P. Foster, Kim Psaila, Kaye Spence, Nadia Badawi, Cathrine Fowler, Virginia Schmied, Charlene Thornton

**Affiliations:** 1 School of Nursing and Midwifery, Western Sydney University, Locked Bag 1797, Penrith, NSW 2751 Australia; 2 Ingham Institute, Liverpool, NSW Australia; 3 Central Clinical School, Discipline of Obstetrics, Gynaecology and Neonatology, University of Sydney, Sydney, NSW Australia; 4 Grace Centre for Newborn Care, The Children’s Hospital at Westmead, Cnr Hawkesbury Road and Hainsworth St, Westmead, NSW 2145 Australia; 5 Sydney Medical School, University of Sydney, Sydney, NSW Australia; 6 Tresillian Chair in Child and Family Health, University of Technology, Broadway, Sydney, NSW 2007 Australia

**Keywords:** gastro-oesophageal reflux, GOR, GORD, mental health, caesarean section, diagnosis

## Abstract

**Background:**

Gastro-oesophageal reflux (GOR) is common in infants. When the condition causes pathological symptoms and/or complications it is considered gastro-oesophageal reflux disease (GORD). It appears to be increasingly diagnosed and causes great distress in the first year of infancy. In New South Wales (NSW), residential parenting services support families with early parenting difficulties. These services report a large number of babies admitted with a label of GOR/GORD. The aim of this study was to explore the maternal and infant characteristics, obstetric interventions, and reasons for clinical reporting of GOR/GORD in NSW in the first 12 months following birth (2000–2011).

**Methods:**

A three phase, mixed method sequential design was used. Phase 1 included a linked data population based study (*n* = 869,188 admitted babies). Phase 2 included a random audit of 326 medical records from admissions to residential parenting centres in NSW (2013). Phase 3 included eight focus groups undertaken with 45 nurses and doctors working in residential parenting centres in NSW.

**Results:**

There were a total of 1,156,020 admissions recorded of babies in the first year following birth, with 11,513 containing a diagnostic code for GOR/GORD (1% of infants admitted to hospitals in the first 12 months following birth). Babies with GOR/GORD were also more likely to be admitted with other disorders such as feeding difficulties, sleep problems, and excessive crying. The mothers of babies admitted with a diagnostic code of GOR/GORD were more likely to be primiparous, Australian born, give birth in a private hospital and have: a psychiatric condition; a preterm or early term infant (37-or-38 weeks); a caesarean section; an admission of the baby to SCN/NICU; and a male infant. Thirty six percent of infants admitted to residential parenting centres in NSW had been given a diagnosis of GOR/GORD. Focus group data revealed two themes: “It is over diagnosed” and “A medical label is a quick fix, but what else could be going on?”

**Conclusions:**

Mothers with a mental health disorder are nearly five times as likely to have a baby admitted with GOR/GORD in the first year after birth. We propose a new way of approaching the GOR/GORD issue that considers the impact of early birth (immaturity), disturbance of the microbiome (caesarean section) and mental health (maternal anxiety in particular).

## Background

Gastro-oesophageal reflux (GOR) is common in preterm and term infants [1] and is usually a self-limiting condition [2]. GOR is generally described as the effortless reflux of gastric contents into the oesophagus and is considered physiologic when the infant thrives and experiences no severe complications [3]. Symptoms may include sleep interruptions [4] frequent spitting up, posseting or vomiting, fussiness during or following feeds and constant or sudden crying, irritability and back arching, and is distressing for infants and stressful for parents especially when regurgitation is frequent [5, 6]. Parents will therefore seek support and education on interventions to help alleviate these symptoms [7].

When the condition causes pathological symptoms and/or complications it is considered to be gastro-oesophageal reflux disease (GORD) [6]. GORD is one of the most commonly misunderstood, and difficult to treat problems that infants experience, and is characterised by chronic symptoms of mucosal damage caused by stomach acid rising from the stomach into the oesophagus [6]. GORD is associated with a range of adverse respiratory, gastrointestinal, and neurobehavioral effects. Adverse effects may include pain (oesophageal and/or ear), wheezing, apnoea, stridor, recurrent bronchiolitis, episodes of oxygen desaturation, aspiration pneumonia, swallowing dysfunction, frequent vomiting, choking and gagging, lower energy intake and excessive weight loss, disorganised and dysfunctional sucking or swallowing, delayed readiness for solid foods or food refusal and delayed development [8, 9]. GORD can cause recurrent sleep interruptions [4, 8, 10, 11] and parental descriptions of the symptoms experienced by their infants, such as an inability to feed and settle can cause considerable parental distress [12, 13]. There may also be differential diagnosis such as hiatus hernia, urinary tract infections, malrotation, pyloric stenosis and cow’s milk intolerance [14].

Transient lower oesophageal sphincter relaxation (TLOSR) resulting in an abrupt drop in oesophageal pressure below gastric pressure, unrelated to swallowing, is regarded as the dominant mechanism and main contributor to the pathophysiology of GORD in both term and preterm infants [1]. The traditional view is that infants with GORD also have delayed gastric emptying, though the role of delayed gastric emptying in promoting GORD is unclear. Gastric emptying time is inversely correlated with gestational age at birth. Preterm babies for example have slower gastric emptying. Gastric emptying has been reported as occuring faster with breastmilk than with formula [15]. It has also been proposed that increased intra-abdominal pressure, and the fact that infants ingest a much higher volume per kilogram of body weight than older children and adults may increase the incidence of reflux during a TLOSR. A baby consuming 180 mL/kg per day corresponds to a daily intake of around 14 L/day in an adult [16]. In addition, term and preterm infants with feeding tubes may experience reflux episodes due to mechanical interference of the lower oesophageal sphincter. It has also been suggested that stiff feeding tubes and wide bore tubes hold open the gastro-oesophageal junction [17].

Determination of the exact prevalence of GOR versus GORD is challenging because there is unclear demarcation between physiologic and pathologic reflux and incidence and prevalence data [18]. In infants 4 to 6 months of age, the prevalence of GOR has been estimated as affecting 23% to 29% of infants in Italy [19] USA [20] and Japan [21] and 41% in Australia [22]. Preterm and low birth weight infants are said to be at particularly high risk of developing GORD [23]) with the overall incidence estimated between 30 and 50% linked to the immaturity of the oesophagus and stomach [16]. This variation in rates shows the difficulty in defining and diagnosing GOR/GORD.

A range of diagnostic investigations may be undertaken in the infant with problematic GORD. The most sensitive objective measure of GORD is the pH probe which provides a quantitative measure of frequency and duration of oesophageal acid exposure [24]. Other methods for detecting GORD include the use of multiple intraluminal impedance and upper gastrointestinal endoscopy and oesophageal biopsy to directly look for inflammation or erosion [25]. Contrast studies can also be used. Most of these investigations for detecting GORD are invasive and should only be directed at infants presenting with recurrent aspiration pneumonia, unexplained apnoeas, non-epileptic seizure-like events and upper airway inflammation [26]. Good quality evidence on pharmacological and non-pharmacological management strategies for GOR and GORD in the infant population is lacking and this creates challenges for clinicians caring for this population [27].

The aim of this study was to explore the maternal and infant characteristics, obstetric interventions, and reasons for clinical reporting of GOR/GORD in NSW in the first 12 months following birth (2000–2011).

## Methods

This study is part of a larger study funded by the Australian Research Council to examine the physical, psychological and demographic characteristics, trends, service needs and co-admissions to other health services of women admitted to residential parenting services (RPS) of Tresillian and Karitane in NSW from 2000 to 2011. There is a tiered system of health services in Australia providing maternal and child health support, including, non-psychiatric day stay and residential parenting services (RPS) such as Tresillian and Karitane (in NSW). RPS provide a range of services and interventions enhance infant caretaking skills and assist adjustment to the work of motherhood [28, 29].

### Research design

A three phase, mixed method sequential design (QUANTITATIVE → Quantitative → Qualitative) design was considered appropriate for this study as it uses a variety of methods to explore complex phenomenon [30]. The philosophical approach chosen for this mixed methods study was pragmatism, which is an approach commonly used in mixed methods research as it challenges the notion of a single, absolute truth being attainable [31]. Pragmatism draws on what works using diverse approaches and valuing both objective and subjective knowledge [32, 33] and is therefore problem centred rather than theory centred with the research question being more important than the method used.

The study was conducted across three sequential phases, each phase informing the next. The sequential design for this study takes a macro (linked data – population wide - quantitative), meso (client notes - RPS - quantitative) and micro (client notes and focus groups with staff - RPS – qualitative) approach. Ethical approval was obtained from the NSW Population and Health Services Research Ethics Committee, Protocol No.2010/12/291. This paper reports the component of the study focused specifically on GOR/GORD. Site specific approval was gained from the two relevant Health Services.

### Phase one

The New South Wales Centre for Health Record Linkage conducted linkage of several datasets via the Health Record Linkage (CHeReL). Hospital admission data – Admitted Patient Data Collection (APDC) was examined for the time period July 1st 2000 till December 31st 2011. The APDC provides demographic and treatment information for all hospital and day stay units within New South Wales (NSW). This dataset was linked to the pregnancy and birth data (mother and baby) NSW, as recorded in the NSW Perinatal Data Collection (PDC). This population based surveillance system contains maternal and infant data on all births of greater than 400 g birth weight and/or 20 completed weeks gestation. The NSW PDC contains data on all births in NSW, around one third of births which occur in Australia annually. Probabilistic data linkage techniques were used for data linkage and de-identified datasets were provided for analysis. Probabilistic record linkage software assigns a ‘linkage weight’ to pairs of records. Records that match perfectly or nearly perfectly on first name, surname, date of birth and address have a high linkage weight, and records matching only on date of birth have a low linkage weight. If the linkage weight is high then it is considered it is likely that the records truly match. If the linkage weight is low it is considered likely that the records are not truly a match. This technique has been shown to have a false positive rate of 0.3% of records [34].

#### Subjects

Infants admitted up to one year of age, recorded in the APDC, who were coded with the International Classification of Diseases (ICD-10-AM) codes K21.0 and K21.9, comprised the cohort of infants with GOR/GORD. Any baby with a congenital abnormality was removed from the cohort in order to eliminate other potential structural defects as a cause of GOR/GORD. The comparison cohort consisted of infants with no ICD-10-AM codes K21.0 and K21.9 documented. Admission events, length of stay and co-morbidities were obtained from the APDC for both the baby and the mother. Co-morbidities for the mother were obtained from diagnostic codes applied to admissions prior to, during and after the birth of the infant who received a GOR/GORD diagnostic code. Data were provided from the PDC and analysed to establish maternal parity, pregnancy events, birth details and neonatal details.

#### Data analysis

Demographic data is reported between the comparison groups according to GOR/GORD diagnosis utilising Chi square for dichotomous variables and mean or median comparison for continuous data. Logistic regression with and without adjustment for maternal and neonatal factors was undertaken. Taking into account the size of the cohort and the number of analyses undertaken, results were considered significant at the level *p* < 0.01. Analysis was undertaken with IBM SPSS v.22®.

### Phase two

The residential parenting services of Tresillian and Karitane in NSW admit around 3400 women a year (3.5% of the population giving birth). Tresillian admits around 2633 women per year in three sites for residential care and Karitane provides residential services for approximately 800 women per year. In order to obtain a more contemporary and detailed understanding of the complex pregnancy and birth factors, particularly psychological, that impact on GORD, in phase two we randomly selected 326 medical records of women admitted to RPS of Tresillian and Karitane in NSW in 2013 across the 12-month period (January 2013 to December 2013); 220 from Tresillian and 106 records from Karitane. Only clinical records from women and infants (women and infants while a dyad in terms of admission have separate files) admitted in the year following birth were examined.

#### Medical record data collection

Coded data collected from client records was entered directly into SPSS with all available variables collected including: demographics of the woman and her infant, type of birth, pregnancy and birth complications, admission to SCN/NICU, postpartum physical health problems and mental health problems and social circumstances and reason for admission. We also collected information about services used /care pathways prior to admission. In this paper we are just reporting the incidence of GOR/GORD reported.

#### Data analysis

The quantitative data were analysed using descriptive statistics and a comparison made between the findings in the linked data and clinical notes for data items that are common to both. The findings from stage one and two were used to inform the focus group questions in stage three. As the high incidence of GOR/GORD emerged from the clinical records review we incorporated this into questions asked during the focus groups.

### Phase three

In phase three, focus groups were used to explore from the perspective of Karitane and Tresillian staff the characteristics of women admitted to RPS, reasons for admission, common prior events and health care pathways, barriers to effective primary and secondary services and any perceived changes in these over the past decade. All staff at Karitane and Tresillian who worked in the RPS were informed about the focus groups at staff meetings and via newsletters and flyers placed in prominent locations.

#### Data collection

In total 45 staff (25 child and family health nurses (CFHN), 10 enrolled nurses/mothercraft nurses, two psychiatrists, six paediatricians and two general practitioners) participated in eight focus groups. The focus groups took approximately one hour and were guided by interview questions/prompts which emerged following analysis of data from previous phases. The issue of GOR/GORD was explored with staff due to the finding in phases one and two of high numbers of babies admitted to RPS who had a GOR/GORD label. All participants agreed to the digital recording of their interviews. Interviews were transcribed verbatim using the transcribing service Pacific Solutions. On receiving the transcripts all identifying information was removed. The full methods around the focus groups are described in another paper [35].

#### Data analysis

Thematic analysis was used to analyse the data. This was undertaken by a research assistant and the first author. Thematic analysis is an iterative process where concepts, categories or themes and relationships with other categories or themes are refined through a series of steps: 1) Multiple readings of the data and listening to the digital recordings to become immersed in the data; 2) Identification and labelling of concepts and development of preliminary themes or categories from these concepts. These themes are captured in phrases, and where appropriate, use the language of the participants; 3) Further coding of the data, identification of linkages and relationships between themes.

#### Integration of the data

Integration of the data describes how the quantitative and qualitative data is mixed during the research process [36]. The data were integrated and analysed at several points throughout this sequential mixed methods study: Data from phase one was used to develop the template for data extraction from the clinical notes in phase two and the data extracted from the clinical notes and linked data (quantitative and qualitative) were used to inform the questions asked during the focus groups with staff in phase three. Once the study was completed further integration occur of the data gathered in all three phases were used to identify common findings and themes and these were used to inform the explanatory conceptual model (Fig. 4).

## Results

### Phase one: Linked data

During the time period there were a total of 1,156,020 admissions recorded in the APDC of infants up to one year of age. Some of these admissions involved multiple admissions for the same infant. Of these admissions, 11,513 (1%) of all admissions contained a diagnostic code for GOR/GORD. This equates to 869,188 individual infants being admitted and of these individual infants, 9152 (1.1%) were admitted with GOR/GORD. The percentage of admissions which included a diagnostic code for GOR/GORD was 1.0%. This figure peaked at 1.1% with a nadir of 0.9%. The number of admissions per infant ranged from 1 to 128. The maternal demographic and birth details are obtained in Table 1.

**Table 1:** Demographic data, pregnancy and birth details mother and baby (up to one year following the birth)

	With GORD *n* = 9152	Without GORD *n* = 860,036	*P*
Age of mother^a^	30.4 (5.50)	30.5 (5.60)	.074
Primiparous	51.4%	41.7%	< 0.000
Mother Australian born	83.0%	70.6%	< 0.000
Gestation at booking visit^a^	9.8 (5.42)	10.9 (6.52)	< 0.000
Born in private hospital	30.5%	24.7%	< 0.000
Any maternal hypertension	9.4%	6.6%	< 0.000
Any maternal diabetes	5.4%	5.3%	0.587
Any maternal smoking	14.0%	13.6%	0.275
Any maternal psychiatric condition	35.0%	9.4%	< 0.000
Multiple birth	8.7%	2.9%	< 0.000
Non-cephalic presentation	8.5%	4.9%	< 0.000
Labour induced	25.7%	25.1%	0.198
Gestation at birth^a^	37.9 (3.10)	39.0 (1.86)	< 0.000
Premature (< 37 weeks gestation)	18.1%	6.4%	< 0.000
Early term birth (37–38 weeks gestation)	27.5%	22.5%	< 0.000
Birth type			
Normal vaginal	50.8%	60.8%	< 0.000
Instrumental	12.0%	10.8%	
Caesarean section	37.2%	28.4%	
APGAR 1^a^	8.1 (1.65)	8.4 (1.36)	< 0.000
APGAR 5^a^	8.9 (0.92)	9.0 (0.73)	< 0.000
Second APGAR< 7	2.9%	1.3%	< 0.000
Birth weight (grams)^a^	3140 (768.0)	3391 (561.2)	< 0.000
Birth weight < 3rd centile	2.3%	1.9%	< 0.000
Admitted to SCN/NICU	29.9%	14.7%	< 0.000
Required resuscitation (any type)	46.9%	36.8%	< 0.000
Intubation of any form	3.6%	0.8%	< 0.000
Male baby	54.6%	50.7%	< 0.000

Chi-square unless other indicated -^a^ (mean and SD)

Women who had a baby with GOR/GORD were more likely to be primiparous, born in Australia, give birth in a private hospital, have hypertension, have a maternal psychiatric condition noted on admission and have undergone a caesarean section. Their babies were more likely to be preterm or early term (37 & 38 weeks), have a birth weight < 3rd centile, be resuscitated at birth or admitted to a SCN/NICU and be male (Table 1.).

**Fig. 1:**
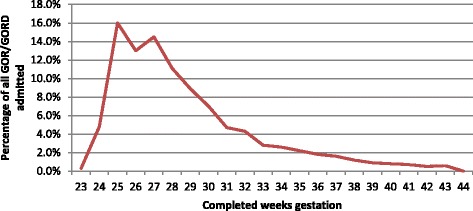
Percentage of infants diagnosed with GOR/GORD by gestational week of their birth

**Fig. 2:**
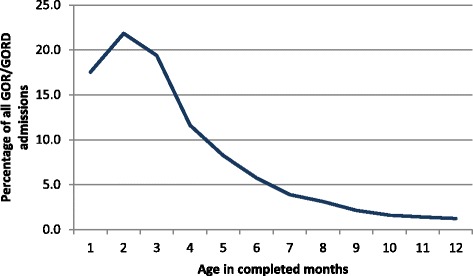
Age in months at first admission expressed as a percentage of all GOR/GORD cases admitted

**Fig. 3:**
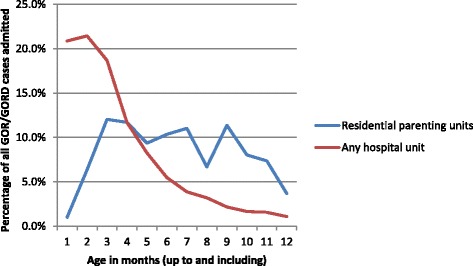
Cases of GOR/GORD admitted to residential parenting units and/or hospital units in first year of life expressed as a percentage of all cases to that unit/s

The highest incidence of GOR/GORD was seen in infants born preterm. GOR/GORD continued to decline for infants born at early gestational ages, levelling out from 40 weeks onwards (Fig. 1.). The first three to four months following the birth was the peak time for admission with GOR/GORD (Fig. 2.), with a notable peak at around six weeks postpartum when the postpartum check is done. In residential units however the numbers of babies with GOR/GORD remained significantly higher and were fairly sustained over the 12 months following birth (Fig. 3.)

Other co-morbidities noted on admission associated with an admission with GOR/GORD were excessive crying, feeding difficulties and sleep disorders which all correlate with reasons for admission to RPS (Table 2).

**Table 2:** Co-morbidities noted on admission with occurrence of > 1% in first year of life

ICD-10-AM code	Definition	% of admissions with GOR/GORD	% of admissions without GOR/GORD	*p*
P07.22	Extreme immaturity 24 or more completed weeks but less than 28 completed weeks	1.1%	0.1%	< 0.000
P07.31	Other preterm infant, 28or more completed weeks but less than 32 completed weeks	1.5%	0.3%	< 0.000
P22.0	Respiratory distress syndrome of newborn	1.6%	1.0%	< 0.000
J21.9	Acute bronchiolitis, unspecified	2.0%	1.05	< 0.000
R06.8	Other and unspecified abnormalities of breathing	3.0%	0.1%	< 0.001
R62.8	Other lack of expected physiological development	3.8%	0.1%	< 0.000
R63.3	Feeding difficulties and mismanagement	9.8%	0.2%	< 0.000
F51.2	Non-organic disorder of the sleep wake schedule	10.2%	0.1%	< 0.000
R68.1	Nonspecific symptoms peculiar to infancy – excessive crying, irritable infant	38.1%	0.6%	< 0.000

Table 3 shows logistic regression results. The following were significantly associated with a diagnostic code of GOR/GORD: 1) Mother being primiparous, born in Australia, giving birth in a private hospital and having a maternal psychiatric condition. 2) Baby being a multiple, born preterm or early term (37 or 38 weeks), born by caesarean section, having a low Apgar, being resuscitated, being intubated, having a NICU or SCN admission and being a male infant.

**Table 3:** Adjusted and unadjusted odds ratios for the development of GOR/GORD (up to 1 year of age) for variables significantly different at cross tabulation

	OR (99% CI)	AOR (99% CI)	*p*
Primiparous	1.48 (1.42–1.54)	1.49 (1.43–1.55)	< 0.001
Mother Australian born	2.03 (1.92–2.15)	1.67 (1.58–1.77)	< 0.001
Born in a private hospital	1.34 (1.28–1.40)	1.45 (1.39–1.52)	< 0.001
Any maternal hypertension	1.47 (1.37–1.57)	1.07 (0.99–1.15)	
Any maternal psychiatric condition	5.20 (4.97–5.43)	4.68 (4.48–4.90)	< 0.001
Multiple birth	3.20 (2.97–3.44)	1.53 (1.40–1.66)	< 0.001
Non-cephalic presentation	1.83 (1.70–1.97)	1.10(1.01–1.19)	< 0.001
Premature (< 37 weeks gestation)	3.24 (3.08–3.43)	1.98 (1.84–2.13)	< 0.001
Early term birth (37–38 weeks)	1.30 (1.25–1.37)	1.37 (1.31–1.45)	< 0.001
Delivered via caesarean section	1.20 (1.17–1.22)	1.13 (1.08–1.78)	< 0.001
Second APGAR < 7	2.18 (1.93–2.47)	0.92 (0.80–1.05)	
Birth weight < 3rd centile	1.21 (1.05–1.39)	0.92 (0.80–1.06)	
Admitted to SCN/NICU	2.48 (2.37–2.60)	1.42 (1.34–1.50)	< 0.001
Required resuscitation (any type)	1.52 (1.45–1.58)	1.07 (1.03–1.12)	< 0.001
Intubation of any form	4.71 (4.21–5.27)	2.46 (1.14–2.82)	< 0.001
Male baby	1.17 (1.12–1.22)	1.14 (1.10–1.14)	< 0.001

The most significant finding was that women with a maternal psychiatric diagnosis were nearly five times as likely to have a baby with GOR/GORD. When the main categories of psychiatric diagnosis were further examined maternal anxiety appeared to have the strongest association with having a baby admitted in the first year following birth with GOR/GORD (Table 4).

**Table 4:** Type of maternal psychiatric diagnosis as a percentage of GOR/GORD admissions

	% of admissions with GOR/GORD	% of admissions without GOR/GORD	*p*
Mental and behavioural disorders due to psychoactive substance abuse (F10-F19)	2.4%	2.4%	0.587
Mood affective disorders (F30-F39)	4.9%	2.1%	< 0.000>
Neurotic, stress related and somatoform disorders (F40-F48)	21.9%	3.7%	< 0.000

### Phase two: Medical records

In the review of 326 medical records we found 36% of infants were reported to have GOR/GORD on admission to the RPS. The rate was 32% in the Tresillian RPS (*n* = 220) and 43% in the Karitane RPS (*n* = 106).

### Phase three: Focus groups

Eight focus groups were undertaken. In total 45 staff took part. There were 25 CFHN, 10 enrolled nurses/mothercraft nurses and 10 doctors (2 psychiatrists, 6 paediatricians and 2 GPs). The average number of years in practice was 17.4 with a mean of 10.7 years working in the RPS. Questions asked in the focus groups included but were not limited to: ‘From your perspective, what are the main reasons for admission to RPS for a mother with an infant under 12 months of age? Have you seen these reasons for admission change in the past decade? Can you describe some of the characteristics of the mothers, their partners and infants that you admit to RPS? ‘In the focus groups GOR/GORD was raised by the participants in six of the groups and mentioned in total 22 times. The paediatricians in particular had the most to say on the subject. The following themes were found: “It is over diagnosed” and “A medical label is a quick fix, but what else could be going on?”

The following quote from a paediatrician summed up the complexity of the situation when it came to GOR/GORD diagnosis and treatment:

*“I probably would say that a lot of babies do have reflux. I would say probably every baby has a form of reflux until they're a bit older. But it's then the interpretation from the parent that they see their baby in pain. My baby's in pain and we've got to do something about it. They go to their GP and they say my baby's very unsettled, my baby's crying a lot. There could be another reason, that she's always in the baby's face, or not allowing the baby down, or not giving it time to settle, or it's overtired as opposed - and the GP just goes, okay, here's the script. He writes you up.” (Paediatrician).*

### It is over-diagnosed

There was a strong feeling from the participants that GOR/GORD was over-diagnosed and that this came from both the medical profession and from parents themselves:

*“I think it gets misdiagnosed a lot too. I don't think every baby that walks through has got GORD” (paediatrician).*

Staff who had been working in the RPS for a while noted that the label of GOR/GORD was being increasingly used.

*“It was very unusual, I guess, 11 years ago, with the reflux medication. Now it just seems every Monday when we do admissions, there's at least two or three, or more, on reflux meds.” (Paediatrician).*

The staff described how women did not want their babies to cry and felt there must be a medical reason if they did. There was also a strong consensus amongst the paediatricians that *“they [doctors] are very quick to medicate”.*

*“They don't want babies to cry ever, so the baby cries, there must be a reason; it must be reflux.”*

*“You have mums that say they go the doctor and say, I want Losec (proton pump inhibitor) and the doctor will write a script.”*

The participants felt that this desire to have a diagnosis, and specialists who tended to over diagnose, was creating a trend where unsettled infants seemed to always be put on anti-reflux medication:

*“I think it's the most over-diagnosed - really the most over-diagnosed disease that we see in infants because everything seems to be down to - it used to be teeth, it used to be child's awake at night because they're teething, now it's everything's that. People like it because they can give medication, the fact that medication does absolutely nothing. They want something to be given, they want to medicalise it. It's sad - it goes right across the industry. It goes - and it's been backed by certain specialists as well, a group of gastroenterologists over there that love it, and it's a real problem. So in fact we see certainly a large majority of the kids that are coming here with unsettled behaviour will come in on anti-reflux [medication]”(Paediatrician).*

*“I've been keeping a tally of how many babies come in on medication for reflux. There's an average of about four out of eight every single week that are either on Zantac, Nexium or Losec, that present. So it's actually really high - a high amount of mums - or a high amount of babies, but I'm just wondering whether that goes back to what you're saying, is that they go to the doctor or paediatrician wanting an answer”(Paediatrician).*

Participants in the focus groups also felt that parents wanted a diagnosis, *“to have a label for their child so that they can feel it's not all my fault.”* The proliferation of organisations and easy access to information through social media was also leading parents to diagnose their own babies.

*“There are reflux associations and groups and bandwagons so people think that sounds exactly like mine, because of course these symptoms are so generic and broad that it really could be anything. But yes my baby cries, yes my baby arches, yes my baby doesn't sleep, yes my baby vomits. If it doesn't vomit it's sounds like reflux anyway. So you can't really - whichever way you look - so I think it's coming from so many areas.”(Paediatrician).*

*“A lot of the time that's what the parents see. So they don't see the other problem. They see the baby crying and the baby vomiting. They look on the internet or talk to parents, other parents, or their friends. Oh, your baby's got reflux. [These are of course] problems but it may not be.”(Nurse).*

### A medical label is a quick fix but what else could be going on?

Participants in the focus groups felt that the medical label of GOR/GORD was often a quick fix that stopped other questions being asked about what else might be going on.

***“*** *Much easier for a parent to feel my baby has a medical cause than maybe I'm not coping. Much easier for a doctor to say it's reflux, I can do something about that but I don't have time to spend an hour asking why your relationship with your mother is so poor that you're not coping and you've got a past history of attachment disorder. So I think it comes both from doctor, I think it comes from expectation of parent, there's media, there's hype, there's a lot of stuff out there about crying babies. You type in crying baby, you see reflux.”(Paediatrician).*

Paediatricians who participated in the focus groups explained the way they tried to reorientate thinking about GOR/GORD in parents who were admitted to the RPS.

*“These are the features I think typically are reflux and these are not - in your baby I'm not seeing this and this and this. I'm not saying there isn't some - you've got to be very diplomatic about who they've seen - but in my experience it's never just reflux. There's always a lot of secondary behaviour.”(Paediatrician).*

There was a feeling that often the real cause of the crying, unsettled baby was not being picked up because of the assumption made that the diagnosis was GORD.

*“Even if the diagnosis is correct, there may be other things operating, making the matters worse. Or sometimes if the diagnosis is wrong - we've had babies here that came in as a feeding problem. That's the other common thing, like breastfeeding problems for example. He's not putting on weight, so the mum's not established breastfeeding well.” (Nurse).*

The nursing staff recognised this was an issue but did not have the authority the paediatricians had to re-orientate thinking around GOR/GORD.

*“We've got a paediatrician that tries to normalise it, and so does cease a lot of the medication if she can” (Nurse).*

### Integrated explanatory conceptual model

**Fig. 4:**
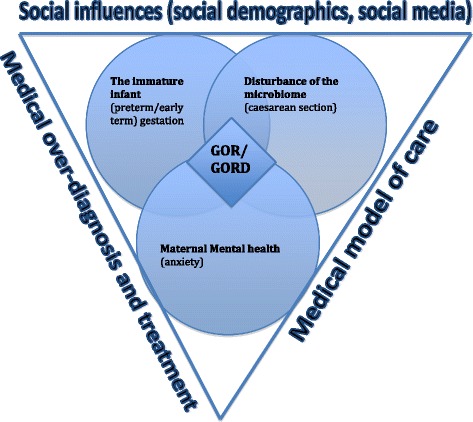
Integrated explanatory conceptual model for GOR/GORD

Based on the research undertaken in this mixed methods study we propose a new way of approaching the GOR/GORD issue that considers the impact of early birth (the immature infant), disturbance of the microbiome (caesarean section) and maternal mental health (anxiety) (Fig. 4).

## Discussion

This mixed methods study aimed to explore the maternal and infant characteristics, obstetric interventions, and reasons for clinical reporting of GOR/GORD in NSW in the first 12 months following birth (2000–2011). The diagnostic code was used for 1% of all infants admitted to hospital in the year following birth. In the RPS however 36% of infants admitted were reported to have GOR/GORD. Babies with GOR/GORD were more likely to be admitted with other disorders such as feeding difficulties, sleep problems and excessive crying, as has been reported in the literature [8–11, 13].

The mothers of babies admitted with a diagnostic code of GOR/GORD were more likely to have a psychiatric condition (especially anxiety), have a preterm or early term infant (37 or 38 weeks), have a caesarean section and have an admission of the baby to SCN/NICU.

The fact that mothers with a mental health disorder are nearly five times as likely to have a baby admitted with GOR/GORD in the first year after birth calls for a re-think about this issue. We propose a new way of approaching the GOR/GORD issue that considers the impact of early birth (the immature infant), disturbance of the microbiome (caesarean section) and maternal mental health (anxiety) (Fig. 4).

### The immature infant

In this study we found a strong association between preterm and early term birth and GOR/GORD. It was not until after 40 weeks that the incidence of GOR/GORD levelled out.

The lower oesophageal sphincter (LES) is made up of intrinsic oesophageal smooth muscle and diaphragmatic skeletal muscle and acts defensively to prevent reflux [37]. Where once preterm infants were thought to have poor LES tone, several studies using manometry documented good LES tone in the preterm and low birthweight populations, which disputes the correlation of prematurity with lower LES tone [37, 38]. Gupta and Jadcherla [39], while evaluating the relationship between segmental oesophageal lengths, growth parameters, gestational age and postmentrual age in preterm and full-term infants, found an increase in the length of the LES increased the length of distal high pressure zone. The authors propose this as a possible mechanism by which GOR/GORD improves with maturation. More recently, however, maturation of LES has been found to be less important in episodes of transient LES relaxations (TLESR) in relation to occurrence of GOR/GORD. TLESRs are abrupt drops in oesophageal pressure below that of gastric pressure, unrelated to swallowing that allow GOR/GORD to occur [40, 41].

#### Iatrogenic immaturity

Intervention during childbirth has escalated dramatically in much of the developed world in the past 20 years [42]. In Australia, late preterm [43](Australian Institute of Health & Welfare, 2015; [44] and early term births [43] have steadily increased over the past decade. Complications of late preterm (34–36 weeks) and early term birth (37–38 weeks) are increasingly being recognised as significant and include increased risk of jaundice [45] and feeding difficulties [46]. In another study using national Australian population data [47] the authors found that among low-risk women who had an unassisted vaginal birth with spontaneous onset of labour and no labour augmentation, the odds of admission to neonatal intensive care or special care nursery were significantly increased when the baby was 37 weeks’ gestation at the time of birth [48] and this remained significant for low risk primiparas who had a baby at 38 weeks gestation.

Some claim that during the final weeks of gestation the fetal brain goes through a marked increase in mass and nerve growth (corticoneurogenesis) which may be best left undisturbed [49]. We have shown that low risk women giving birth in private hospitals in NSW are much more likely to give birth at earlier gestations than their public hospital counterparts for every week up to and including 40 weeks [42]. The finding in this study that early term birth and birth in a private hospital is associated with an increase in GOR/GORD may be due to several interacting factors. Very few women who book care with a private obstetrician in a private hospital have psychosocial screening done that might detect and enable mental health issues to be addressed. Secondly, the numbers of early term deliveries due to increased intervention is much higher than in the public sector. The fact that this group of women are generally more educated and access health services more readily may also lead to an increased chance of diagnosis or over-diagnosis. As was identified in the focus groups paediatricians and general practitioners may more readily label a crying baby as having GOR then delving into other possible underlying factors that would require longer appointment time frames.

### Disturbance of the microbiome

The fact that GOR/GORD was associated with caesarean birth and resuscitation/admission to SCN/NICU provides another interesting possible answer to this complex issue. Research on impact of mode of birth and antibiotic use on the infant microbiome is gaining importance. While we could not identify antibiotic usage in this study, Australian research has shown that nearly half of all babies that go to neonatal units will have antibiotics administered [50].

Evidence on the potential risks associated with the use of antibiotics (both given to the mother during pregnancy/labour and birth and to the baby after birth), includes increased rates of asthma in early childhood [51, 52], infant allergies to cow’s milk [53]; and higher rates of obesity [54].

There is mounting evidence that babies born by caesarean delivery have different gut microbiota in the first months of life to those born vaginally. This suggests the route of birth may be fundamental to the founding physiology of the gut flora. The CHILD study from Canada used DNA sequencing to detect microbes in faecal samples from infants at age four months and found those born by caesarean delivery had low bacterial richness and diversity compared to those born vaginally [55].

Following caesarean delivery there are higher numbers of cells secreting immunoglobulins (ImmunoglobulinA and ImmunoglobulinG) at one year of age, and some studies have found increased rates of asthma, gastroenteritis, rhinitis, food allergies, and type 1 diabetes in babies born via caesarean section [56–58]. Antibiotic use has also been shown to alter microbiota especially when used with caesarean section and changes have been seen in children up to 12 months of age, especially where babies are not breastfed [59]. We did not have reliable enough data to look at method of infant feeding which is a limitation of our study.

Recent studies indicate a crucial role of the intestinal microbiota in the pathogenesis of gastrointestinal disorders [60]. Probiotics have been found to significantly increase intestinal blood flow [61], gastric emptying rate and improve feeding tolerance [62] and growth [63]. Probiotics may play a crucial role in the modulation of intestinal inflammation and they have been found to be effective in several randomised controlled trials in reducing regurgitation episodes in preterm and term infants [62, 64–66].

### Maternal mental health

The fact that having a maternal admission with a mental health disorder increases the risk of a diagnosis in the infant by more than four times is a significant finding that has not previously been identified. Maternal anxiety appears to be the most influential factor.

The ambiguous presenting symptoms, the serious sequela of GOR/GORD combined with an inability to diagnosis the disease inadvertently leads to over diagnosis of GOR/GORD as was identified in this study and this in turn increases the anxiety of parents. It may be that inconsistent parenting by anxious, inexperienced mothers increases infant crying. The fact that primiparous women were more likely to have an infant with GOR/GORD supports this.

The literature demonstrates that the maternal infant dyad is particularly at risk in the context of excessive crying. Several studies have investigated the relation between excessive crying and maternal depression [67–69], with more recent literature highlighting the effect of maternal trait anxiety/psychological distress and anxiety disorders during pregnancy on excessive infant crying and emotional problems in childhood [70]. Petzoldt, et al., [71] found maternal anxiety prior to pregnancy was associated with an increased risk for excessive crying; even when adjusting for maternal depression. Maternal anxiety may lead to intrusiveness that possibly intensifies infant crying. The association of maternal anxiety disorders and excessive crying was found to be increased when associated with anxiety provoking incidents occurring during the peri-partum [71].

Excessive crying, feeding and sleeping problems have been associated with caregiver-infant interactional problems, a disturbed caregiver–infant relationship, and severe parental impairment and frustration [72, 73]. Mothers of infants with feeding difficulties may become anxious, leading to feelings of failure and fear of rejection by the infant. Maternal feeding anxiety are reported to be common in GOR/GORD [74]. The developmental origins of health and disease hypothesis which suggests early environmental factors influence mental and physical health into adulthood has been described [75, 76]. Persistent early regulatory problems (excessive crying > 3 months of age, feeding and/or sleeping difficulties) have been reported as early markers for subsequent unfavourable childhood outcomes such as Attention Deficit Hyperactive Disorder (ADHD) [76].

### Towards a new understanding of GOR/GORD

In this study paediatricians and nurses working in the RPS identified an over diagnosis of GOR/GORD which might lead to other underlying causes and factors not being addressed. When feeding back the results of the study to the paediatricians involved in the focus groups they identified that the peak in GOR/GORD diagnosis was around the routine six week postnatal check and indicated this was an another example of possible service provider influence when it came to diagnosis of GOR/GORD (Fig. 2).

A major issue in the over diagnosis of GOR as GORD is the inability or unwillingness of health professionals to differentiate between GOR and GORD. GOR in infants is a normal physiological process. The symptoms of feeding difficulty, sleeping problems and excessive crying while usually transient during infancy, may persist into early childhood [76]. All infants cry in the first few weeks of life, however more than 25% infants cry > 2 h day. Unfortunately some parents may find it difficult to differentiate between what is normal and abnormal infant behaviour. Healthy infants have regurgitation that is physiologic resolving without intervention in 95% of the individuals by 12–14 months of age [77, 78]. Feeding difficulties, sleeping problems and excessive infant crying are the most frequent reasons for parents to consult a doctor [79].

In a study aimed at evaluating the implementation of the North American Society for Pediatric Gastroenterology, Heapatology, and Nutrition (NASPGHAN) guidelines [2] for treatment of GOR, Quitadamo et al., [80] found GORD was diagnosed based on clinical symptoms irrespective of the age of the child and that 39% of general paediatricians prescribe proton pump inhibitors’ (PPIs) in infants with unexplained crying and/or distressed behaviour and in infants with uncomplicated recurrent regurgitation and vomiting. Notably PPIs have been found to be no better than using a placebo in crying babies in the first few months of life [81, 82]. In addition, there is no simple, reliable and accurate method for the diagnosis of GORD [26]. Intra-oesophageal pH monitoring and multichannel intraluminal impedance and manometry, separately or in combination, although the most commonly used investigations for GORD correlate poorly with symptoms and are not reliable diagnostic tools in the infant population [83]. As a consequence normal regurgitation and normal crying, or abnormal crying due to a cause other than GORD, may be mistaken for GORD.

Anxious or inexperienced parents may link infant fussiness and crying to their own feelings of inadequate parenting [84]. They fear the existence of some sinister cause for their infant’s discomfort so seek out a medical rationale for the infant’s continued unsettled behaviour [85]. Parents faced with prolonged infant crying, may find their physical and psychological resources stretched to the extreme. We know the parent–child relationship can have lasting effects on the healthy development of the child [84]. In addition, parental partner relationship quality may suffer as it is closely related to the well-being of the baby. In light of the adverse impact of infant crying on the family and infant, and the associated treatment costs, prevention of such problems is a priority.

Future research could focus on active normalising of GOR for parents through a discussion which emphasises that reflux rarely requires further investigation or treatment. The National Institute of Clinical Excellence (NICE) guidelines [26] offer recommendations for the management of the infant with GOR which feature the stepwise trialling of management strategies to reduce symptoms of GOR and the avoidance of routine investigation or treatment for GORD until overt signs of GORD exist [26]. Notably there are no references to psychological and emotional support for parents included in the guidelines despite the negative effects of adverse infant behaviour being well documented [86–89]. This needs to be an urgent focus of future research. Health care providers such as midwives, neonatal nurses and child and family health nurses may be better placed to normalise infant behaviour and reduce over diagnosis. They also spend more time with parents where conversations can occur that elicit other possible factors.

### Limitations

There are several limitations in this study. The advantages of using population-based datasets and linkage to other databases include the size of the sample and the high accuracy of a validated dataset. Limitations include the restricted number of variables included and the limited specific information on potential influencing variables. The ICD-10-AM codes are also only from hospital admissions and so will underestimate the babies treated for GOR/GORD at a community level. In addition clinical coders will look for a diagnoses of GOR/GORD in the medical records. If a clinician gave the diagnosis then the record would be coded for that, or if it was in the history of the patient. The inclusion of the diagnosis by a clinician in the notes does not mean it is not necessarily confirmed by test results. A small number of cases with a low linkage rate (0.3%) were not included meaning that there is the possibility of missing variables. Previous validation studies have shown high levels of data accuracy for most of the diagnoses and procedures conducted during labour and delivery in the state-wide data base [45, 46]. However, the recording of medical conditions and smoking tend to be underreported [45, 47]. There are several other socio-demographic factors we could not control for, including education and income that may provide valuable insight into associated socio-demographics. This study can only provide an overview of possible associations with GOR/GORD and does not imply causality. We also do not know on what basis infants were given a GORD diagnosis as opposed to GOR and so we have combined these. This makes it also really hard to determine the real prevalence and burden of the problem. The medical records reviewed in this study were from one year only but the random sampling increased the reliability. The focus groups were only undertaken with staff in the NSW RPS and so may not be representative of views of health professionals in other services or outside of NSW.

## Conclusions

We propose a new way of approaching the GOR/GORD issue that considers the impact of early birth (immaturity), disturbance of the microbiome (caesarean section) and mental health (maternal anxiety in particular). The current approach of treating a crying baby with anti-reflux medication may not get to the root cause and therefore will not address underlying issues leading to the problem.

## Abbreviations

ADHD: Attention Deficit Hyperactive Disorder
APDC: Admitted Patient Data Collection
CFHN: Child & Family Health Nurses
GOR: Gastro Oesophageal Reflux
GORD: Gastro Oesophageal Reflux Disease
LES: Lower oesophageal sphincter
NICE: National Institute of Clinical Excellence
NICU: Neonatal Intensive Care Unit
NSW: New South Wales
PCD: Perinatal Data Collection
PPI: Proton Pump Inhibitors
RPS: Residential Parenting Services
SCN: Special Care Nursery
TLOSR: Transient lower oesophageal sphincter relaxation

## References

[1] Omari T, Barnett C, Benninga M, Lontis R, Goodchild L, Haslam R, Dent J, Davidson G (2002). Mechanisms of gastro-oesophageal reflux in preterm and term infants with reflux disease. Gut.

[2] Vandenplas Y, Rudolph CD (2009). Pediatric gastroesophageal reflux clinical practice guidelines: joint recommendations of the north American Society for Pediatric Gastroenterology, hepatology, and nutrition (NASPGHAN) and the European Society for Pediatric Gastroenterology, hepatology, and nutrition (ESPGHAN). J Pediatr Gastroenterol Nutr.

[3] Salvatore S, Vandenplas Y (2002). Gastroesophageal reflux and cows milk allergy: is there a link?. Pediatrics.

[4] Machado R, Woodley FW, Skaggs B, Di Lorenzo C, Splaingard M, Mousa H (2013). Gastroesophageal relux causing sleep interruptions in infants. J Pediatr Gasttoenterology Nutr.

[5] Willmott A, Murphy MS (2004). Gastro-esophageal reflux. Curr Paediatr.

[6] Vandenplas Y, Rudolph CD. Pediatric Gasroesophageal reflux clinical practice guidelines: joint recommendations of the north American Society for Pediatric Gastroenterology, Heapatology, and nutrition (NASPGHAN) and the European Society for Peditric Gstroenterology, Heaptology, and nutrition (ESPGHAN). J Pediatr Gastroenterol Nutr. 2009;49(498–547) 10.1097/MPG.0b013e3181b7f563 19745761

[7] Neu M, Corwin E, Lareau SC, Marcheggiani-Howard C (2012). A review of nonsurgical treatment for the symptom of irritability in infants with GERD. J Spec Pediatr Nursing.

[8] Ammari M, Djeddi D, Leke A, Delanaud S, Stephan-Blanchard E, Bach V, Telliez F (2012). Relationship between sleep and acid gastroe-oesophageal reflux in neonates. J Sleep Res.

[9] Field D, Garland M, Williams K (2003). Correlates of specific childhood feeding problems. J Pediatr Child Health.

[10] Hawdon JM, Beauregard N, Slattery J, Kennedy G (2000). Identification of neonates at risk of developing feeding problems in infancy. Dev Med Child Neurol.

[11] Woodley FW, Hayes J, Mousa H (2009). Acid gastroesophageal reflux in symptomatic infants is primarily a function of classic 2-phase and pH-only acid reflux event types. J Pediatr Gastroenterol Nutr.

[12] Heine RG, Jordan B, Lubitz L, Meehan M, Catto-Smith AG (2006). Clinical predictors of pathological gastro-oesophageal reflux in infants with persistent distress. J Pediatr Child Health.

[13] Vandenplas Y, Hassall E (2002). Mechanisms of gastrooesophageal reflux and gastroesophageal reflux disease. J Pediatr Gastroenterol Nutr.

[14] Dogra H, Lad B, Sirisena D (2011). Paediatric gastro-oesophageal reflux disease. British J Med Pract.

[15] Ramirez A, Wong W, Shulman R (2006). Factors regulating gastric emptying in preterm infants. J Pediatr.

[16] Orenstein: Regurgitation & GERD. . J Pediatr Gastroenterol Nutr 2002, 32:S16–S18. 10.1097/00005176-200104001-00008 11321410

[17] Peter CS, Wiechers C, Bohnhorst B, Silny J, Poets CF (2002). Influence of nasogastric tubes on gastroesophageal reflux in preterm infants: a multiple intraluminal impedance study. J Pediatr.

[18] Eisen G (2001). The epidemiology of gastroesophageal reflux disease: what we know and what we need to know. Eisen GM.

[19] Iacono G, Merolla R, D'Amico D, Bonci E, Cavataio F, DP L, Scalici C, Indinnimeo L, Averna MR, Carroccio A (2005). Gastrointestinal symptoms in infancy: a population-based prospective study. Dig Liver Dis.

[20] Nelson SP, Chen EH, Syniar GM, Christoffel KK: Prevalence of symptoms of gastroesophageal reflux during infancy. A pediatric practice-based survey. In: *Archives of Pediatric and Adolescent Medicine.* Edited by group. PPR, vol. 151; 1997: 569–572. 10.1001/archpedi.1997.02170430035007 9193240

[21] Miyazawa R, Tomomasa T, Kaneko H, Tachibana A, Ogawa T, Morikawa A (2002). Prevalence of gastro-esophageal reflux-related symptoms in Japanese infants. Pediatr Int.

[22] Martin M, Pratt N, Kennedy D, Ryan P, Ruffin R, Miles H, Marley J (2002). Natural history and familial relationships of infant spilling to 9 years of age. Pediatrics.

[23] DiPietro JA, Cusson RM, O’Brien Caughy M, Fox NA: Behavioral and physiologic effects of non-nutritive sucking during gavage feeding in preterm infants**.** Pediatr Res 1994, 236(2):207–2014. 10.1203/00006450-199408000-00012 7970936

[24] Wenzi T, Silny J, Schenke S, Peschgens T, Heimann G, Skopnik H (1999). Gastroesophageal reflux and respiratory phenomena in infants: status of the intraluminal impedance technique. J Pediatr Gastroenterolo Nutrition.

[25] DeVault KR, Castell DO (2005). Updated guidelines for the diagnosis and treatment of gastroesophageal reflux disease. Am J Gastroenterol.

[26] NICE: Gastro-oesdophageal reflux disease in children and young people:NICE guideline 1 :Methods, evidence and recommendations. In*.* Edited by Excellence NIfHaC: National Collaborating Centre for Women’s and Children’s Health; 2015.

[27] Psaila K, Foster J, Rajendram R, Preedy VR, Patel VB (2015). Critically Ill Infants with Gastro-Oesophageal Reflux. *diet and Nutrition in Critical Care. Volume 1*, edn.

[28] Fisher J, Rowe H: Building an Evidence Base for Practice in Early Parenting Centers. A Systematic Review of the Literature and a Report of an Outcome Study. In*.* Melbourne: Key Centre for Women’s Health in Society, School of Population Health, University of Melbourne; 2004.; 2004.

[29] Rowe HJ, Fisher JRW. The contribution of Australian residential early parenting centres to comprehensive mental health care for mothers of infants: evidence from a prospective study. Int J Ment Heal Syst. 2010;4. 10.1186/1752-4458-4-6 PMC2873569 20380739

[30] Cresswell J, Plano Clark V, Cresswell J, Plano Clark V (2011). Choosing a Mixed Methods Design. *Designing and Conducting Mixed Methods Research.* 2nd edn.

[31] Johnson RB, Onwuegbuzie AJ (2004). Mixed methods research: a research paradigm whose time has come. Educ Res.

[32] Shannon-Baker P. Making paradigms meaningful in mixed methods research. J Mixed Methods Res. 2015:1–16.

[33] Feilzer MY (2010). Doing mixed methods research pragmatically: implications for the rediscovery ofPragmatism as a research paradigm. J Mixed Methods Res.

[34] Centre for Health Record Linkage: Centre for Health Record Linkage, “Quality Assurance Report,“2012, http://www.cherel.org.au/media/24160/qa report 2012.pdf. 2014.

[35] Fowler C, Schmied V, Dickinson M, Dahen HG (2016). Working with complexity: experiences of caring for mothers seeking residential parenting services in new South Wales, Australia. J Clin Nurs.

[36] Creswell J, Klassen A, Plano Clark V, Clegg Smith K: Best practices for mixed methods research in the health sciences in*.* Edited by Research OoBaSS. New England: National Institute of Health 2011.

[37] Newell SJ, Sarkar PK, Durbin GM, Booth IW, McNeish AS (1988). Maturation of the lower oesophageal sphincter in the preterm baby. Gut.

[38] Boix-Ochoa J, Canals J (1976). Maturation of the lower esophagus. J Pediatr Surg.

[39] Gupta A, Jadcherla SR. The Relationship Between Somatic Growth and In Vivo Esophageal Segmental and Sphincteric Growth in Human Neonates. 2006;43(1):35–41. 10.1097/01.mpg.0000226368.24332.50 PMC4028631 16819375

[40] Pena EM, Parks VN, Peng J, Fernandez SA, Di Lorenzo C, Shaker R, Jadcherla SR. Lower esophageal sphincter relaxation reflex kinetics: effects of peristaltic reflexes and maturation in human premature neonates. Am J Physiol Gastrointest Liver Physiol. 2010;23 10.1152/ajpgi.00289.2010 PMC3006240 20864655

[41] Kawahara H, Dent J, Davidson G (1997). Mechanisms responsible for gastroesophageal reflux in children. Gastroenterology.

[42] Dahlen H, Tracy S, Tracy MB, Bisits A, Brown C, Thornton C: Rates of obstetric intervention and associated perinatal mortality and morbidity among low-risk women giving birth in private and public hospitals in NSW (2000–2008): a linked data population-based cohort study. BMJ Open, 2014**;**4**:**e004551**.**10.1136/bmjopen-2013-004551. 10.1136/bmjopen-2013-004551 PMC4039844 24848087

[43] Welfare. AIoH: Australia's mothers and babies 2013-in brief. In: *Perinatal statistics series.* Canberra: AIHW; 2015.

[44] Cheong JL, Doyle LW (2012). Increasing rates of prematurity and epidemiology of late preterm birth. J Paediatr Child Health.

[45] Bhutani VK, Stark AR, Lazzeroni LC, Poland R, Gourley GR, Kazmierczak S, Meloy L, Burgos AE, Hall JY, Stevenson DK (2013). Predischarge screening for severe neonatal hyperbilirubinemia identifies infants who need phototherapy. J Pediatr.

[46] Reddy UM, Ko CW, Willinger M (2006). Early term births (37–38 weeks) are associated with increased mortality. Am J Obstet Gynecol.

[47] Tracy S, Tracy M, Sullivan E (2007). Admission of term infants to neonatal intensive care: a population-based study. Birth.

[48] Tracy SK, Tracy MB, Sullivan E (2007). Admission of term infants to neonatal intensive care: a population-based study. Birth.

[49] Adams-Chapman I (2009). Insults to the developing brain and impact on neurode- velopmental outcome. Journal of communication disorders. J Commun Disord.

[50] Osowicki J, Gewee A, Noronha J, Britton PN, Isaacs D, Lai TB, Nourse C, Aven M, Moriarty P, Francis JR (2015). Australia-wide point prevalence survey of antimicrobial prescribing in neonatal units how much and how good?. Pediatr Infect Dis J.

[51] Stensballe LG, Simonsen J, Jensen SM, Bonnelykke K, Bisgaard H (2013). Use of Antibiotics during Pregnancy Increases the Risk of Asthma in Early Childhood. J Pediatr.

[52] Collier CH, Risnes K, Norwitz ER, Bracken MB, Illuzzi JL: Maternal infection in pregnancy and risk of asthma in offspring. Maternal Child Health Journal 2013, doi 10.1007/s10995–013–1220-2. 10.1007/s10995-013-1220-2 23338127

[53] Metsälä J, Lundqvist A, Virta LJ, Kaila M, Gissler M, Virtanen SM (2013). Mother’s and Offspring’s use of antibiotics and infant allergy to Cow’s milk. Epidemiology.

[54] Ajslev TA, Andersen CS, Gamborg M, Sørensen TIA, Jess T (2011). Childhood overweight after establishment of the gut microbiota: the role of delivery mode, pre-pregnancy weight and early administration of antibiotics. Int J Obes.

[55] Azad MB, Konya T, Maughan H, Guttman DS, Field CJ, Chari RS, Sears MR, Becker AB, Scott JA, Kozyrskyj AL et al: Gut microbiota of healthy Canadian infants: profiles by mode of delivery and infant diet at 4 months. Can Med Assoc J 2013**.** doi:10.1503 /cmaj.121189. 10.1503/cmaj.121189 PMC3602254 23401405

[56] Cardwell CR, Stene LC, Joner G, Cinek O, Svensson J, Goldacre MJ, et al. Caesarean section is associated with an increased risk of childhood-onset type 1 diabetes mellitus: a meta-analysis of observational studies. Diabetologia. 2008;51(726–35) 10.1007/s00125-008-0941-z 18292986

[57] Thavagnanam S, Fleming J, Bromley A, Shields MD, Cardwell CR (2008). A meta-analysis of the association between Caesarean section and childhood asthma. Clin Exp Allergy.

[58] Hyde MJ, Mostyn A, Modi N, Kemp PR (2012). The health implications of birth by caesarean section. Biol Rev.

[59] Azad MB, Konya T, Persaud RR, Guttman DS, Chari RS, Field CJ, Sears MR, Mandhane PJ, Turvey SE, Subbarao P et al: Impact of maternal intrapartum antibiotics, method of birth and breastfeeding on gut microbiota during the first year of life: a prospective cohort study. BJOG 2015, Online Sept 2015 (doi: 10.1111/1471-0528.13601). 10.1111/1471-0528.13601 26412384

[60] Indrio F, Di Mauro A, Riezzo G, Civardi E, Intini C, Corvaglia L, Ballardini E, Bisceglia M, Cinquetti M, Brazzoduro E (2014). Prophylactic use of probiotic in the prevention of colic, regurgitation, and functional constipation: a randomized clinical trial. JAMA Pediatr.

[61] Havranek T, Al-Hosni M, Armbrecht E (2013). Probiotics supplementation increases intestinal blood flow velocity in extremely preterm infants. J Perinatol.

[62] Indrio F, Riezzo G, Raimondi F, Bisceglia M, Cavallo L, Francavilla R (2008). The effects of probiotics on feeding tolerance, bowel habits, and gastrointestinal motility in preterm newborns. J Pediatr.

[63] Yamasaki C, Totsu S, Uchiyama A, Nakanishi H, Masumoto K, Washio Y, Shuri K, Ishida S, Imai K, Kusuda S (2012). Effect of bifidobacterium administration on very-low-birthweight infants. Pediatr Int.

[64] Garofoli F, Civardi E, Indrio F, Mazzuchelli I, Angelini M, Tinelli C, Stronati M (2014). The early administration of lactobacillus reuteri DSM 17938 controls regurgitation episodes in full-term breastfed infants. Int J Food Sci Nutr.

[65] Indrio F, Di Mauro A, Riezzo G, Civardi E, Intini C, Corvaglia L, Ballardini E, Bisceglia M, Cinquetti M, Brazzoduro E (2014). JAMA Pediatr.

[66] Indrio F, Reizzo G, Raimondi F, Bisceglia M, Filannino A, Cavallo L, Francavilla R (2011). Lactobacillus reuteri accelerates gastric emptying and improves regurgitation in infants. Eur J Clin Investig.

[67] Christl B, Reilly N, Smith M, Sims DG, Chavasse F, Austin MP (2013). The mental health of mothers of unsettled infants: is there value in routine psychosocial assessment in this context?. Arch Womens Mental Health.

[68] McMahon C, Barnett B, Kowalenko N, Tennant C, Don N (2001). Postnatal depression, anxiety and unsettled infant behaviour. Aust N Z J Psychiatry.

[69] Radesky JS, Zuckerman B, Siverstein M, Rivara FP, Barr M, Taylor JA, Lengua LJ, Barr RG (2013). Inconsolable infant crying and maternal postpartum epressive symptoms. Pediatrics.

[70] Petzoldt J, Wittchen HU, Einsle F, Martini J. Maternal anxiety versus depressive disorders: specific relations to infants’ crying, feeding and sleeping problems. Child Care Health Dev. 2015; 10.1111/cch.12292 26490836

[71] Petzoldt J (2014). Maternal anxiety disorders predict excessive infant crying: a prospective longitudinal study. Arch Dis Child.

[72] Postert C, Averbeck-Holocher M, Arhtergarde S, Muller JM, Furniss T. Regulatory disorders in early childhood: correlates in child behavior, parent–child relationship, and parental mental health. Infant Mental Health Journal. 2013, 33:173–86. 10.1002/imhj.20338 28520094

[73] Bolten M (2013). Infant psychiatric disorders. Eur Child Adolesc Psychiatry.

[74] Karacetin G, Demir T, Erkan T, Cokugras FC, Sonmez AB (2011). Maternal psychopathology and psychomotor development of children with GERD. JPGN.

[75] Vaiserman AM (2015). The developmental origins of health and disease hypothesis which suggests early environmental factors influence mental and physical health into adulthood has been described. Dev Dyn.

[76] Schmid G, Wolke D (2014). Preschool regulatory problems and attention-deficit/hyperactivity and cognitive deficits at school age in children born at risk: different phenotypes of dysregulation?. Early Hum Dev.

[77] Hegar B, Dewanti NR, Kadim M, Alatas S, Firmansyah A, Vandenplas Y (2009). Natural evolution of regurgitation in healthy infants. Acta Paediatr.

[78] Fike F, Mortellaro VE, Pettiford MP, Ostlie DJ, SD SP (2011). Diagnosis of gastroesophageal reflux disease in infants. Pediatr Surg Int.

[79] Lightdale JR, Gremse DA (2013). Gastroesophageal reflux: management guidance for the pediatrician. Pediatrics.

[80] Quitadamo P, Papadopoulou A, Wenzl T, Urbonas V, Kneepkens CMF, Roman E, Orel R, Pavkov DJ, Dias JA, Vandenplas Y (2014). European pediatricians’ approach to children with GER symptoms: survey of the implementation of 2009 NASPGHAN-ESPGHAN guidelines. J Pediatr Gastroenterol Nutr.

[81] Bialek-Gieruszczak D, Konarska Z, Skorka A. No effect of proton pump inhibitors on crying and irritability in infants: systematic review of randomized controlled trials. J Pediatr. 2015;166(767–70) 10.1016/j.jpeds.2014.11.030 25556017

[82] Orenstein SR, Hassall E, Furmaga-Jablonska W, Atkinson S, Raanan M. Multi-centre, double-blind randomized placebo-controlled trial assessing the efficacy of proton pump inhibitor lansoprazole in infants with symptoms of gastroesophageal reflux disease. J Pediatr. 2009;154(514–520) 10.1016/j.jpeds.2008.09.054 19054529

[83] van der Pol R, Smits MJ, Venmans L, Boluyt N, Benninga MA, Tabbers MM (2013). Diagnostic accuracy of tests in pediatric gastroesophageal reflux disease. J Pediatr.

[84] Verhage ML, Oosterman M, Schuengel C (2015). The linkage between infant negative temperament and parenting self-efficacy: the role of resilience against negative performance feedback. Br J Dev Psychol.

[85] Lightdale JR, Gremse DA (2013). Section on gastroenterology, hepatology and nutrition. Gastroesophageal relux: management guidance for the pediatrician. Pediatrics.

[86] Verhage ML, Oosterman M, Schuengel C (2015). The linkage between infant negative temperament and parenting self-efficacy: The role of resilience against negative performance feedback. British J Dev Psychol.

[87] Meijer AM, van den Wittenboer GLH (2007). Contribution of infants’ sleep and crying to marital relationships of first-time parent couples in the 1st year after childbirth. J Fam Psychol.

[88] Jordan B, Heine RG, Meehan M, Catto-Smith AG, Lubitz L (2006). Effect of antireflux medication, placebo and infant mental health intervention on persistent crying: a randomized clinical trial. J Paediatr Child Health.

[89] Petzoldt J, Wittchen HU, Einsle F, Martini J: Maternal anxiety versus depressive disorders: specific relations to infants‚ Äô crying, feeding and sleeping problems. Child Care Health Dev 2015. 10.1111/cch.12292 26490836

